# Palmprint Based Multidimensional Fuzzy Vault Scheme

**DOI:** 10.1155/2014/819031

**Published:** 2014-04-16

**Authors:** Hailun Liu, Dongmei Sun, Ke Xiong, Zhengding Qiu

**Affiliations:** ^1^School of Computer & Information Technology, Beijing Jiaotong University, Beijing 100044, China; ^2^Beijing Key Laboratory of Advanced Information Science and Network Technology, Beijing 100044, China

## Abstract

Fuzzy vault scheme (FVS) is one of the most popular biometric cryptosystems for biometric template protection. However, error correcting code (ECC) proposed in FVS is not appropriate to deal with real-valued biometric intraclass variances. In this paper, we propose a multidimensional fuzzy vault scheme (MDFVS) in which a general subspace error-tolerant mechanism is designed and embedded into FVS to handle intraclass variances. Palmprint is one of the most important biometrics; to protect palmprint templates; a palmprint based MDFVS implementation is also presented. Experimental results show that the proposed scheme not only can deal with intraclass variances effectively but also could maintain the accuracy and meanwhile enhance security.

## 1. Introduction

Biometric based authentication taking physiological or behavioral characteristics of an individual, such as fingerprint, palmprint, face, and voice, for personal identification is an enhanced authentication mechanism. However, such authentication technology needs large-scale capture and storage of biometric data which leads to serious concern about leaking of privacy and identity theft. Unlike password or token based authentication, biometric characteristics are inherent to a person; once compromised, it would never be reissued or refreshed.

Biometric cryptosystems [1] combining cryptography with biometrics offering provable security are considered to be a promising solution to above issues.

For a biometric cryptosystem, helper data (also named secure sketch [2] in some literatures) is generated and stored instead of biometric template in enrolment phase. Without the genuine user's biometric traits, the helper data do not leak any information about the original templates. In authentication, helper data is used to help regenerate the key. The key can be used for identification or directly used in cryptosystems.

Many famous biometric cryptosystems have been proposed such as fuzzy commitment scheme [3], fuzzy extractor [4], and fuzzy vault scheme (FVS) [5]. Among these schemes, the fuzzy vault scheme proposed by Juels and Sudan [5] has become one of the most popular key-binding approaches, because it provides effective and provable security for biometric template protection [6]. Since the FVS is proposed, many biometric characteristics have been used to construct biometric cryptosystems based on FVS, such as fingerprints [7], iris [8], and face [9].

In essence, FVS is a secret-sharing mechanism. A user uses unordered set *A* to encrypt the secret to generate the vault. A large number of chaff points are added into the vault to protect valid data. If another set *B* provided by another user is close enough to set *A*, the protected secret can be decoded successfully, where Reed-Solomon code is introduced to correct variances between two sets.

By applying FVS to design biometric template protection scheme, the most important aspect is dealing with large intraclass variances which are inherent to biometric features due to changing collecting environments such as changes of pose or illumination. However, as some researchers point out [8, 9], special use of the Reed-Solomon code in the fuzzy vault scheme is not appropriate, which results in much extra work to reduce the intraclass variances, such as Nandakumar et al. [7] who proposed using high curvature points extracted from the fingerprint orientation field as helper data to align the template and query minutiae, Lee et al. [8] who proposed a shift-matching method for iris alignment, and Liu et al. [10] who proposed a 3D fuzzy vault to improve the variance handling ability.

The issues lie in that the fuzzy vault scheme is designed for set differences which is often used to measure the similarity of two sets in finite field [11], while Euclidian distance measuring two sets in real number field is often used for similarity measure in biometric techniques. Inspired by the similarity between template matching in biometric recognition and valid point filtration from fuzzy vault, in this paper, we propose a more general scheme named multidimensional fuzzy vault scheme (MDFVS) to deal with real-valued intraclass variances.

The contributions of this paper are as follows:

1. a concrete construction of MDFVS is proposed. To construct variance-tolerant space, first, a metric space is defined. Second, the input feature vector is mapped into subvectors to construct a linear subspace in which intraclass variances can be tolerated. Last, the vault locking and unlocking algorithms based on such linear subspace are given;
2. palmprint based MDFVS implementation is presented. In the implementation, classic linear discriminant analysis (LDA) is used to extract feature vector from palmprints for validating the feasibility of MDFVS. Experimental results in terms of receiver operating characteristic (ROC) curves and curve of genuine accept rate (GAR) versus number of chaff vectors are given and discussed.

The rest of this paper is organized as follows.  Section 2 presents the framework of MDFVS and its locking and unlocking algorithms. The implementation of palmprint based MDFVS is presented in Section 3.  Section 4 discusses the experimental results. We summarize our works in Section 5.

## 2. Multidimensional Fuzzy Vault Scheme

In this section, the concrete construct of MDFVS is elaborated. First, the vault and operating space is defined. Second, the locking and unlocking algorithms are presented in detail.

### 2.1. Definition of Vault and Operating Field

The proposed vault *V* is composed of many vectors *V*_*i*_ with the same length; that is, *V* = {*V*_*i*_}_*i*=1_^*M*^, where *V*_*i*_ = [*v*_*i*1_, *v*_*i*2_,…, *v*_*ij*_,…, *v*_*in*_], *i* = 1,2,…, *M*, *n* is the length of the vector, and *M* is the total number of vectors in vault. The vector in the vault generated from biometric features is named as genuine vectors, and the vectors generated randomly are named as chaff vectors.

If elements in the vector are from real number field *R* and Euclidean metric is used to measure the similarity between two vectors, the metric space becomes a linear space [12]. The proposed vault locking and unlocking algorithms below are operating on such linear space.

### 2.2. Locking Algorithm

The vault locking algorithm is described in Algorithm 1. The input and output parameters are as follows.

**Input**: Parameters *k*, *t*, and *r* such that *k* ≤ *t* ≤ *r*; a cryptographic key *κ* ∈ GF^SL^(2) and biometric feature vector FV ∈ *R* ^*n*^.

**Output**: A vector set *V*.

In the input parameters, *k* represents the degree of the polynomial to be constructed, *t* represents the number of genuine vectors in the vault which is derived from biometric feature vector FV, and *r* represents the total number of vectors in the vault including genuine and chaff vectors.

A cryptographic key *κ* ∈ GF^SL^(2) is a bit string with length SL. Biometric feature vector FV ∈ *R* ^*n*^ is a real-valued feature vector extracted from biometric traits using specific feature extraction method.

There are two important steps in the locking algorithm: feature vector mapping and genuine vector generation.

*(1) Feature Vector Mapping*. Enrolling feature vector FV is mapped into *t* subvectors, that is, {*fv*_*i*_}_*i*=1_^*t*^. These subvectors construct a linear space. This linear space is named as variance-tolerant space since Euclidean distance can be applied to measure the similarity between two vectors in this linear space. Even though there are variances, the genuine vector can also be recognized by similarity measurement as long as the query subvector is close enough to the concealed genuine vector.

Feature vector mapping is used for embedding variance-tolerant linear space into fuzzy vault. The variance-tolerant capability is determined by the mapping method. 

*(2) Genuine Vector Generation*. Feature data in a subvector can be used to evaluate the polynomial. Given the subvector set {*fv*_*i*_}_*i*=1_^*t*^ obtained in step (1), single feature data *f*_*i*_ is selected from each subvector *fv*_*i*_ for evaluating the polynomial *p* to get the point set {(*f*_*i*_, *p*(*f*_*i*_))}_*i*=1_^*t*^. Because the feature data *f*_*i*_ is already contained in the subvector *fv*_*i*_, we pad the *p*(*f*_*i*_) at the end of *fv*_*i*_ to form the final vector (*fv*_*i*_, *p*(*f*_*i*_)) for the vault. The padded vector (*fv*_*i*_, *p*(*f*_*i*_)) is called genuine vector. Totally, there are *t* genuine vectors that are generated.

### 2.3. Unlocking Algorithm

The vault unlocking algorithm is described in Algorithm 2. The input and output parameters are as follows.

**Input:** query feature vector FV′, the vault *V*, and parameter triple (*k*, *t*, *r*).

**Output:** bit string *κ*′ ∈ GF^SL^(2) ∪ *ϕ*.

The main step of vault unlocking algorithm is genuine vector filtering.

Based on different algebraic number field that elements in the vectors belong to, the distance computation can be Hamming metric, Euclidean metric, set differences, and so on. In the proposed vault unlocking algorithm, elements in vectors are from real number field and Euclidean metric is used to measure the distances between vectors; the genuine vector recognition can be rewritten as shown in Algorithm 3.

The genuine vector filtering is carried out between subvector {*fv*_*i*_′}_*i*=1_^*t*^ and the vault {*v*_*j*_}_*j*=1_^*r*^. For given query subvector *fv*_*i*_′, we compute the distances between *fv*_*i*_′ and each vector in *V*. There are *r* − *i* + 1 distances that were computed. The vector in *V* corresponding to the minimum distance is considered as the genuine vector. And then these filtered genuine vectors are concentrated to form a long vector LV. If the distance between FV′ and LV is greater than a given threshold *T*, the vault unlocking fails. Otherwise, pairwise data are extracted from recognized vectors for polynomial reconstruction, that is, (*x*_*i*_, *y*_*i*_) ← *v*_*i*_.

Given degree *k* and points set *Q*, a polynomial *p*′ can be reconstructed. If all points in *Q* are genuine points, the original polynomial can be reconstructed accurately, and the key can be recovered successfully from the coefficients of the reconstructed polynomial.

## 3. Implementation of Palmprint Based MDFVS

Palmprint is one of the most important biometrics, from which many unique features such as principal lines and wrinkles can be extracted for personal identification [13]. However, the current palmprint identification systems are still not secure enough due to many potential attacks and privacy leaking threats [14]. In this section, we introduce a secure palmprint identification system based on proposed MDFVS.

First, the framework of palmprint based MDFVS is illustrated. Second, the implementation of locking and unlocking algorithms of palmprint based MDFVS is elaborated. In the interpretation of the two algorithms, we focus on the implementations.

### 3.1. Framework of Proposed Palmprint Based MDFVS Implementation

**Figure 1:**
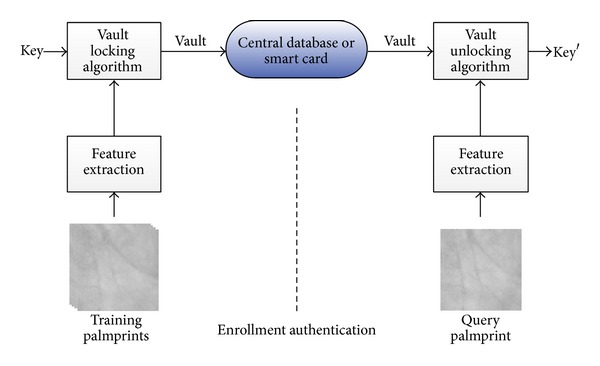
Framework of palmprint based MDFVS.

The proposed framework of palmprint based MDFVS is shown in Figure 1.

There are two inputs for the enrollment: randomly generated key and training palmprints. The output is the vault. In authentication, the vault is retrieved and taken as the input of the unlock algorithm. If the extracted feature vector from query palmprint is close enough to the enrolled one in terms of Euclidean distance, the protected key can be regenerated correctly.

### 3.2. Locking Palmprint Based MDFVS

**Figure 2:**
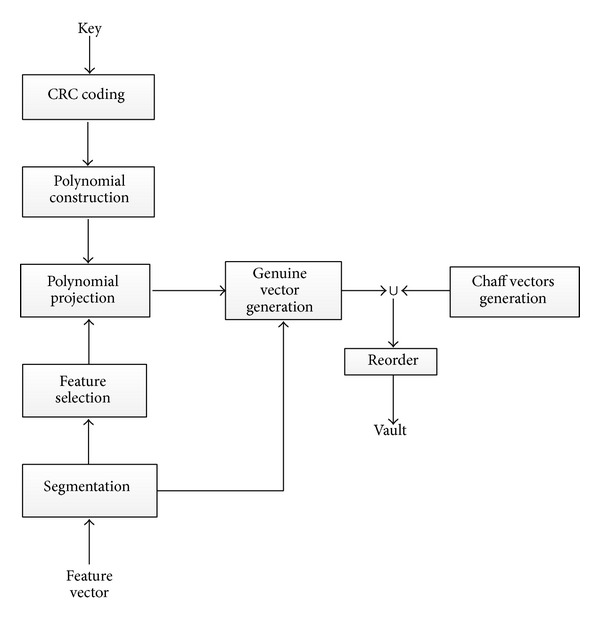
Flowchart of vault locking.

The flowchart of implementation of vault locking is shown in Figure 2. In the following, three main modules including polynomial construction, genuine vector generation by polynomial projection, and vault generation are described in detail.

*(1) Polynomial Construction*. The key is transformed to 16 × *n* bits by zero padding method. 16-bit CRC-16 code [15] is generated for error checking. By appending the 16-bit CRC code to the end of the key, a (16 + 1) × *n*-bit new key is obtained. The new key is segmented into *n* + 1 segments. Each segment is regarded as binary representation of a 16-bit number. There are totally *n* + 1 numbers: *c*_0_, *c*_1_,…, *c*_*n*_.

Because a polynomial with degree greater than eight is very difficult to be reconstructed accurately, the highest degree of polynomials we recommend is eight. If (*n* + 1) is greater than nine, multiple eight-degree polynomials can be constructed based on these 16-bit numbers, for example, *p*_1_(*x*) = *c*_0_ + ⋯+*c*_8_ *x* ^8^; *p*_2_(*x*) = *c*_9_ + ⋯+*c*_17_ *x* ^8^; ….

*(2) Genuine Vector Generation by Jointing Projected Values*. To construct the variance-tolerant linear subspace, we simply segment the feature vector into subvectors with same length. An appropriate feature {*x*_*i*_} is selected from subvectors for polynomial projection. After polynomial projection, the evaluated value {*p*_*j*_(*x*_*i*_)} is appended to the end of the subvector from which the *x*_*i*_ is selected.

**Figure 3:**
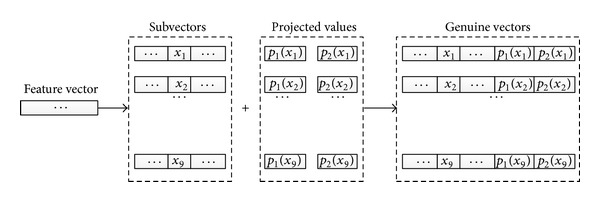
Genuine vector generation by jointing evaluations of polynomials.

For example, there are two polynomials to be constructed, *p*_1_(*x*) and *p*_2_(*x*). The number of subvectors after segmentation is nine. If nine features are selected from nine subvectors, respectively, for polynomial projection (selecting one feature from each subvector), the diagrams of genuine vector generation are shown in Figure 3. 

*(3) Vault Generation*. The chaff vectors are generated randomly. Since the ranges of normalized features and projected values are in different value range, the random generated elements in chaff vector with indexes corresponding to features are normalized to the value range of normalized features, and the elements in chaff vectors with indexes corresponding to projected values are normalized to the value range of projected values.

After normalization, chaff vectors are combined with genuine vectors to form the vault. After combination, the vectors in the union are sorted in ascending order based on the values of the first columns, and then the vault is stored in central database or smart card.

### 3.3. Palmprint Based MDFVS Unlocking

**Figure 4:**
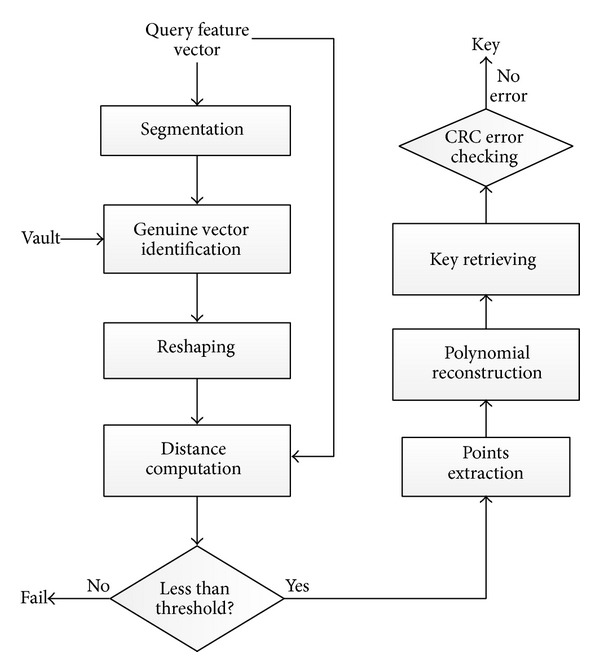
Flowchart of vault unlocking.

The flowchart of vault unlocking implementation is shown in Figure 4.

Two main modules, genuine vectors filtering and key recovering, are described as follows.

*(1) Genuine Vectors Filtering from Vault*. First, the query feature vector is segmented into subvectors in the same way used in vault locking.

Given a query subvector *V*_*i*_, Euclidean distance between *V*_*i*_ and all vectors in the vault is computed.

Because polynomial projection values were appended at the end of genuine vectors, the length of genuine vector is longer than the query subvector. In distance computation, the padded polynomial projection values are removed so as to keep the same length for Euclidean distance computation. The vector in vault corresponding to the minimum distance is considered as genuine vector.

After genuine vectors filtering, these recognized vectors are reshaped to form a single longer vector, in which all polynomial projection values are discarded. The Euclidean distance between reshaped vector and query feature vector is computed and compared with the predetermined threshold. If the distance is less than the predetermined threshold, the unlocking fails; otherwise, the pairwise data for polynomial reconstruction are extracted from the filtered genuine vectors. 

*(2) Key Recovering by Polynomial Reconstruction*. One or more polynomials are reconstructed by Lagrange interpolation [16] based on extracted point set. The coefficients of constructed polynomials are concatenated for CRC error detection. If no error is detected, the recovered key would be the same as the original one with probability 1.

## 4. Experimental Results

### 4.1. Database and Feature Extraction

The palmprint database used in our experiments is handmetric authentication Beijing Jiao Tong University database (HA-BJTU) [17], in which there are 1973 hand images of 98 users, each image is captured using digital camera. There are two collecting sessions: 5 samples of each user were captured for the first time, and, 2 months later, the rest of samples were captured. The region of interest (ROI) which is named as palmprint is extracted for experiments. The size of the ROI image is sampled to 128∗128. For each user, 5 palmprint are used as training samples for feature extraction, and the left 1483 palmprints are used for test.

The classic feature extraction algorithm LDA is used to extract the features from palmprints. Because there are 98 users in the database, a feature vector with 97 coefficients is extracted to represent each palmprint image.

### 4.2. Accuracy Evaluation of Proposed System

Genuine accept rate (GAR) and false accept rate (FAR) are used to evaluate the accuracy of the proposed implementation. The GAR is defined as the percentage that the protected key was regenerated accurately when genuine users attempted to obtain the key using his or her palmprints and registered vault. The number of genuine attempts is 1483 in our experiments. The FAR is defined as the success rate that a genuine user's key was regenerated when an imposter attempted to steal any key using his or her palmprints and the genuine user's vault. Impostor attempts were simulated via unlocking a user's vault using palmprints of all other users. The times of imposter attempts in our experiments are 1483 × (98 − 1) = 143851.

**Figure 5:**
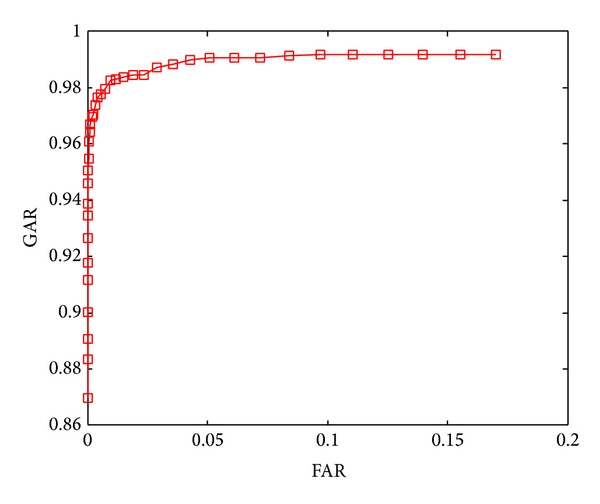
ROC curve when segmentation is 2 × 48.

**Figure 6:**
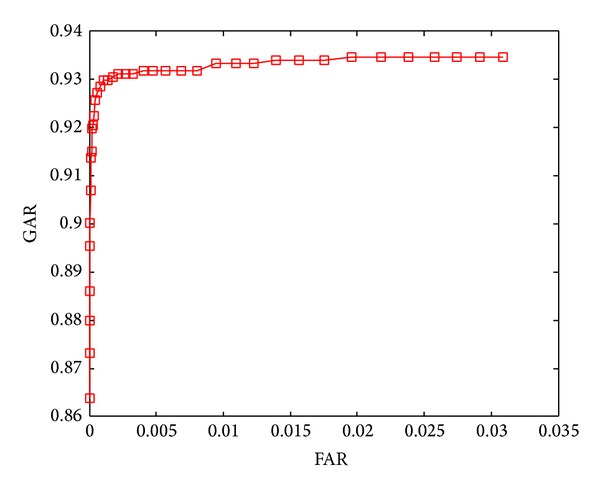
ROC curve when segmentation is 4 × 24.

**Figure 7:**
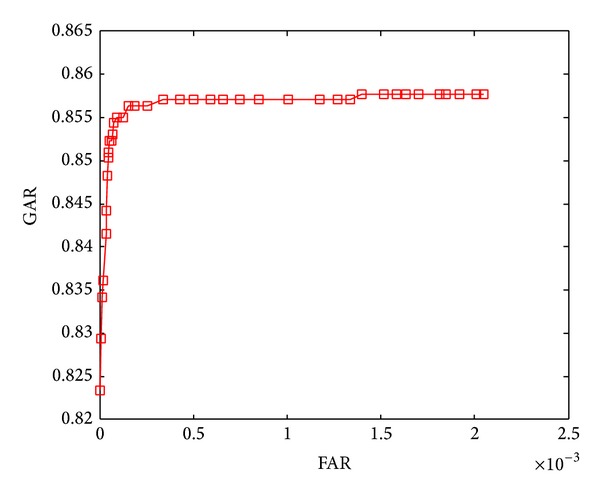
ROC curve when segmentation is 6 × 16.

**Figure 8:**
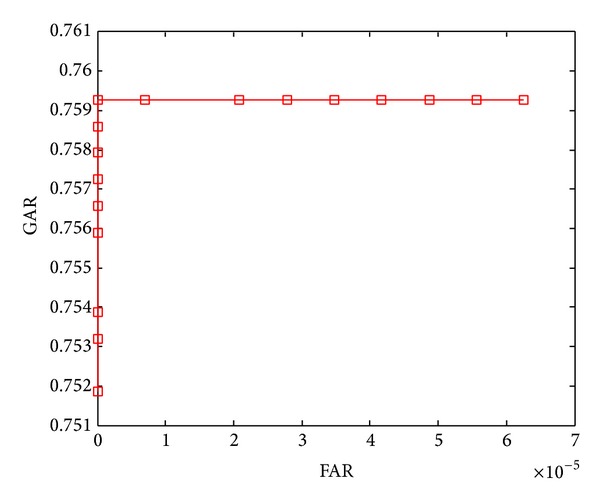
ROC curve when segmentation is 8 × 12.

The ROC curves shown in Figures 5–8 are obtained through varying thresholds. Four curves are corresponding to four kinds of subspace constructions by segmenting LDA feature vector. These segmentations are shown in Table 1. 800 chaff vectors are added to the vault.

From the four ROC curves we can see the following.

The GARs decrease with the increase of segments. Because the length of LDA feature vector is constant, more segments mean less features in each genuine vector, which leads to weaker capability of resisting disturbance taken in by chaff vectors in genuine vector identification. At last, the lower accuracy of genuine vectors filtering results in lower GAR.

With the increase of segments, The FARs decrease too. With the decreasing number of features in each genuine vector, the disturbances taken in by chaff vectors become stronger. For an imposter, it becomes more difficult to distinguish genuine and chaff vectors, so the FARs decrease.

### 4.3. Security Analysis

We consider the security of proposed system under the brute force attack assuming that the attacker has accessed the database and gotten the vault.

To compute the complexity of the brute force attack that is finding enough genuine vectors from the vault for polynomial reconstructions, the min-entropy which was proposed by Dodis et al. [4] is used in our work.

The min-entropy of filtering a genuine vector set GV which contains enough genuine vectors for vault unlocking from vault *V*, can be simplified as follows [18]:

(1) $$\begin{array}{c} H_{\infty }\left(\text{GV}\;|\;V\right)=-\log\left(\frac{C_{t}^{k+1}}{C_{r}^{k+1}}\right), \end{array}$$

where *r* is the total number of vectors in the vault, *t* is the number of genuine vectors in the vault, and *k* is the degree of the polynomial.

In our experiments, to unlock the vault successfully, all genuine vectors are required to be found out from the vault. So the expression of the min-entropy can be rewritten as follows:

(2) $$\begin{array}{c} H_{\infty }\left(\text{GV}\;|\;V\right)=-\log\left(\frac{1}{C_{r}^{t}}\right). \end{array}$$

With different segmentations, the number of genuine vectors *t* is different. In our experiments, *t* are 2, 4, 6, and 8, respectively, and *r* = 800 + *t*. The resulted security bits are 18.3 bits, 34.0 bits, 48.4 bits, and 61.9 bits, respectively.

**Figure 9:**
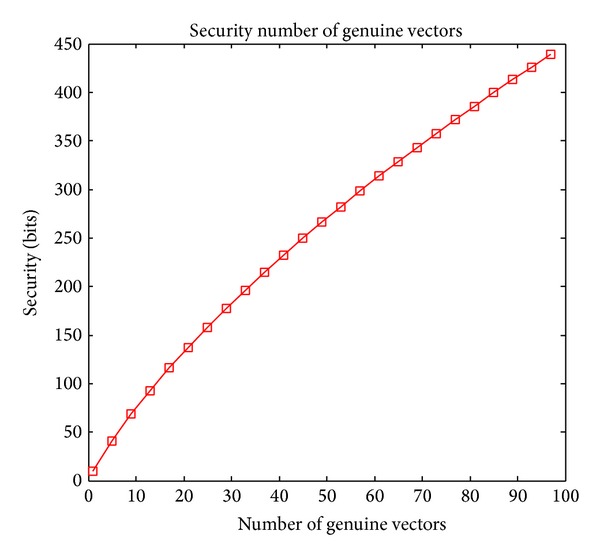
Security with different number of genuine vectors.

When the number of chaff vectors is fixed, the relation between the security and the number of genuine vectors is reflected in Figure 9. From the figure, we can see that, by increasing the number of genuine vectors that is required to be filtered out for vault unlocking, the security increases rapidly. However, more genuine vectors required to be filtered out for polynomial reconstruction means higher error probability of genuine vector filtration, which will decrease the system accuracy. To enhance the security, an alternative way is to increase another variable, for instance, the number of chaff vectors, which will be discussed in the following section.

### 4.4. GAR versus the Number of Chaff Vectors

Given constant number of genuine vectors, the security of the fuzzy vault system only depends on the number of chaff vectors in the vault. More chaff vectors means higher security. For traditional fuzzy vault scheme, large number of chaff points will cause great interferences in filtration of genuine points, which would decrease the GAR of the system. For the proposed MDFVS in this paper, this tradeoff can be alleviated.

**Figure 10:**
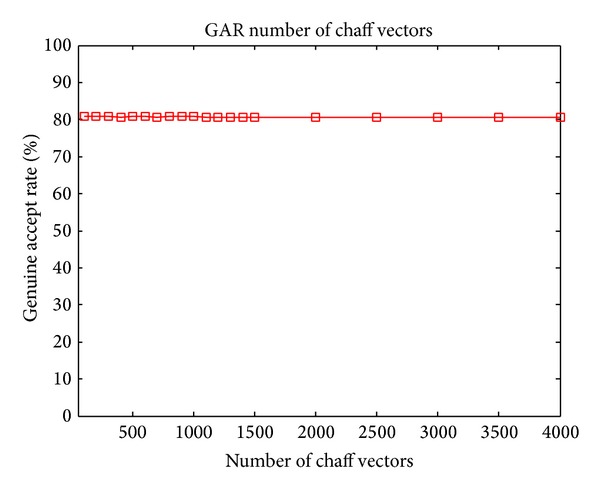
GAR versus number of chaff vectors under segmentation 7 × 13.

The relation between GAR and the number of chaff vectors of proposed MDFVS is shown in Figure 10. From the figure, we can see that, with the increase of the number of chaff vectors, the GAR is very stable. The tradeoff issue is solved. The reason lies in that one randomly generated element in chaff vector may be close to the feature data in genuine vector with the same index, but the probability is very small that all randomly generated elements in chaff vector are close to the corresponding features in genuine vector. But the features in legal query feature vector and genuine vector are always close to each other. Consequently, the filtration of genuine vectors is less affected and the GAR can be maintained. This means that the proposed MDFVS can maintain the system accuracy while enhancing the system security by adding more chaff vectors.

## 5. Conclusions

Since ECC used in traditional FVS is not appropriate to handle real-valued biometric intraclass variances, we have proposed a new scheme named multidimensional fuzzy vault scheme, in which a new error-tolerant mechanism was designed. Given traditional FVS is suitable for protecting point-set based biometric features such as minutiae of fingerprint, the proposed MDFVS is suitable for protecting real-valued biometric feature vectors. Palmprint based MDFVS implementation was also designed in this paper. Experiments based on classic LDA feature vector demonstrated the validity of proposed MDFVS in terms of accuracy and security.

## Figures and Tables

**Algorithm 1:**
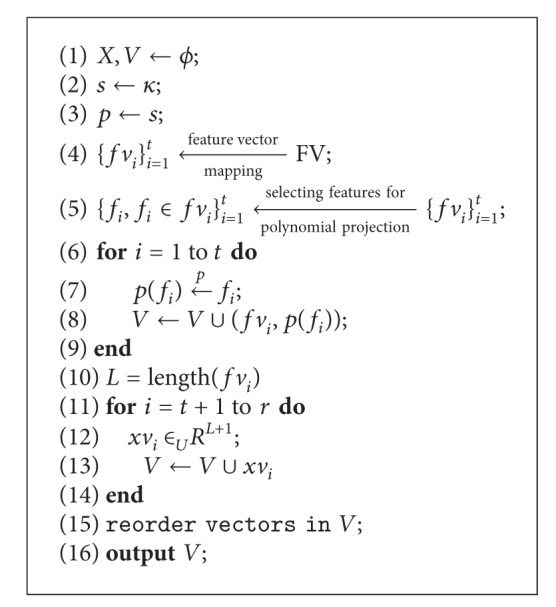
Locking vault.

**Algorithm 2:**
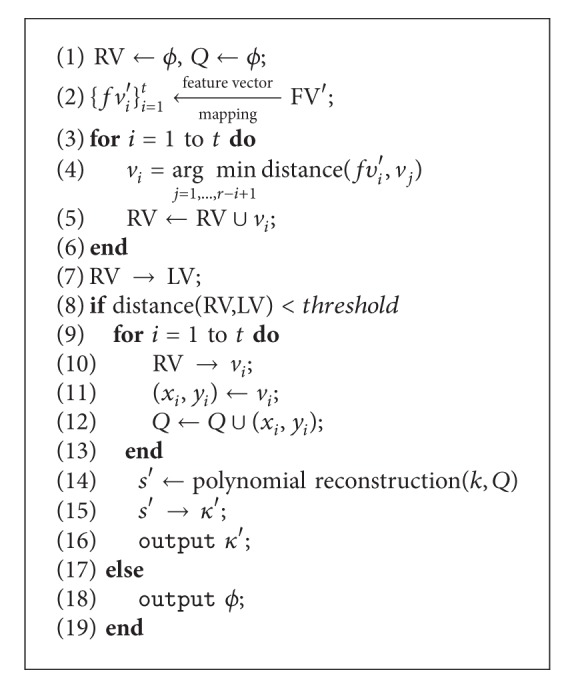
Unlocking vault.

**Algorithm 3:**
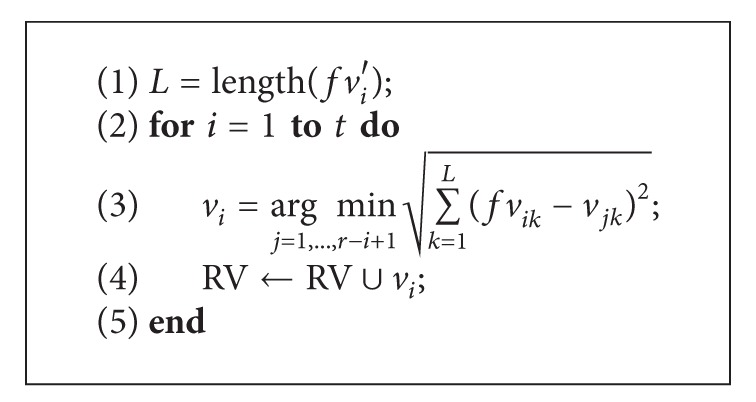
Genuine vector filtering.

**Table 1:** Segmentations of feature vector.

ROC curve in	Number of subvectors	Length of subvectors
Figure 5	2	48
Figure 6	4	24
Figure 7	6	16
Figure 8	8	12
